# Acute occupational exposures reported to the Dutch Poisons Information Center: a prospective study on the root causes of incidents at the workplace

**DOI:** 10.1186/s12995-022-00360-4

**Published:** 2022-09-05

**Authors:** Anja P. G. Wijnands, Irma de Vries, Tim Verbruggen, Maxim P. Carlier, Dylan W. de Lange, Saskia J. Rietjens

**Affiliations:** 1 Dutch Poisons Information Center, University Medical Center Utrecht, Utrecht University, PO Box 85500, 3508 GA Utrecht, the Netherlands; 2 Department of Intensive Care Medicine, University Medical Center Utrecht, Utrecht University, PO Box 85500, 3508 GA Utrecht, the Netherlands

**Keywords:** Acute occupational intoxications, Hazardous substances, Root causes, Poison Control Center, Preventive measures

## Abstract

**Background:**

Hazardous substances at the workplace can cause a wide variety of occupational incidents. This study aimed to investigate the nature and circumstances of acute occupational intoxications reported to the Dutch Poisons Information Center.

**Methods:**

During a one-year prospective study, data on the circumstances and causes of the incident, the exposure(s) and clinical course, were collected by a telephone survey with victims of an acute occupational intoxication.

**Results:**

We interviewed 310 patients. Most incidents occurred in industry (25%), building and installation industry (14%) and agriculture (10%). Patients were often exposed via multiple routes (ocular contact 40%, inhalation 34% and dermal contact 33%). Acids and alkalis were often involved. Exposure often occurred during cleaning activities (33%). The main root causes of these accidents were: technical factors such as damaged packaging (24%) and defective apparatus (10%), organizational factors such as lack of work instructions (44%) and poor communication or planning (31%), and personal factors such as disregarding work instructions (13%), not (adequately) using personal protective equipment (12%) and personal circumstances (50%) such as inaccuracy, time pressure or fatigue. The majority of the patients only reported mild health effects and recovered quickly (77% within 1 week).

**Conclusions:**

Poison Center data on occupational exposures provide an additional source of knowledge and an important basis for poisoning prevention strategies related to hazardous substances at the workplace. These data are useful in deciding which risk mitigation measures are most needed in preventing future workplace injuries.

**Supplementary Information:**

The online version contains supplementary material available at 10.1186/s12995-022-00360-4.

## Background

The Dutch Poisons Information Center (DPIC) offers expert advice to health care professionals on the diagnosis and treatment of poisoned patients. In 2019, the DPIC received almost 35,000 telephonic enquiries on individuals exposed to a wide variety of substances. Less than 3% of the enquiries involved acute exposures to hazardous substances at work [1]. The number of acute occupational intoxications reported to the DPIC more than doubled, from 375 in 2015 to 871 in 2019. This increase was much larger than the rise in total DPIC consultations, which was approximately 5% over the same time period [2]. Data reported by the Dutch Labor Inspectorate and the Dutch Injury Surveillance system, show that each year about 1000 patients visit an Emergency Department because of an accident at work involving “exposure to a chemical” or “poisoning.” In contrast to our data, the reported number of these type of incidents is rather stable from 2016 to 2019 [3, 4].

The increase in the number of occupational intoxications reported to our Poison Center triggered us to set up the present prospective study in order to gain more detailed information about the nature and circumstances of occupational intoxications. We hypothesize that a Poisons Center can collect valuable data and make a distinction between various causal factors at the level of pure technical/mechanical failures during the work process, the organization of the company’s work activities, and personal factors related to individual workers. Such a distinction may reveal relevant information and guidance for the development of specific preventive measures and worker training programs.

## Methods

All acute occupational intoxications reported to the DPIC between 1 and 9-2020 and 1-9-2021 were included. Chronic occupational exposures were excluded. The consulting health care professionals were informed on the possible clinical effects and treatments according to the standard DPIC procedure. At this stage, the patient’s identity was unknown to the DPIC, except for sex, age, and bodyweight. Subsequently, the physician was asked to inform their patient(s) about the study. Patients were separately included in case of incidents with more than one victim. Only after patient agreement, physicians provided the DPIC with the patients’ contact information (identifiable data were omitted before analysis). A case was considered lost-to follow-up (LFU) if there were no contact details available, the patient was not reachable or the patient did not wish to participate in the study.

Patients who agreed to participate, were interviewed within 2 weeks by telephone. Before the interview, informed consent was obtained by telephone (and voice recorded) after information was provided on the content, duration and confidentiality of the interview and the anonymous processing of the data.

A standardized questionnaire was used with questions on the circumstances of the incident, the exposure(s) (e.g. products involved, route), the clinical course (e.g. health effects, recovery-time) and treatment (e.g. first-aid treatments, use of healthcare). Causal factors were investigated at a level of: 1. technical factors (e.g. defective machinery); 2. organizational factors (e.g. availability of work instructions); 3. personal factors (e.g. fatigue, time pressure, actual use of personal protective equipment (PPE)). This classification was derived from the EU-OSHA hierarchy of prevention and control measures [5].

The standardized questionnaire consisted of a combination of open and multiple choice questions (for more information see Additional file 1). The duration of the interview was approximately 15 min. In case health effects were still present during the interview, the patient was contacted a second time, mostly within 3 weeks.

Data were registered in Castor EDC, a cloud based Data management platform. Anonymity of the patients was warranted by allocating serial numbers to each questionnaire. A separate file in Excel v16.0 (Microsoft, Redmond, USA) was used to link the serial numbers to the information in the DPIC database. Calculations and data analysis were performed in Excel v16.0 (Microsoft, Redmond, USA) and SPSS v26 (IBM, Armonk, USA). Descriptive statistics (percentage, median, interquartile range (IQR), full range) were used to provide an overview of patient and exposure characteristics, root causes, clinical course and treatment. Descriptive data on root causes were used to identify risk factors for occupational exposures. Pearson’s Chi-squared tests were used to test statistical differences in demographics between patients with follow-up and patients LFU and to test if there was a relation between the main root causes and specific business classes.

The accredited Medical Research Ethics Committee of the University Medical Center Utrecht considered the Dutch Medical Research Involving Human Subjects Act not applicable to this study.

## Results

### Occupational intoxications reported to the DPIC

From September 1st 2020 to August 31st 2021, 924 cases of presumed occupational intoxications were reported to the DPIC. Thirty cases did not meet the inclusion criteria and were excluded. Of the 894 included cases, 310 patients (34.7%) were interviewed. Five hundred eighty-four patients (65.3%) could not be interviewed and were considered LFU. The most important reasons for LFU were missing contact information (46.6%), refusal to participate (25.7%) and not reachable by phone (18.2%). There were no demographic differences between the FU and LFU group.

All results in the following paragraphs relate to the interviewed patients (*n* = 310, FU group). The interviewed patients were mainly males (71.6%) with a median age of 38 years (IQR: 19 years, full range: 16–63 years). The DPIC was mostly consulted by general practitioners (78.7%), and to a lesser extent by patients themselves (5.5%), emergency department staff (5.2%), hospital doctors (4.8%), ambulance workers (3.5%) and other medical professionals (2.3%).

### Industries, type of employment and nature of activities

In Table 1 a summary is given of the various business classes (industries) in which the patients were working. The list is compiled according to the Standard Business Classification List of Statistics Netherlands (CBS) [6].

**Table 1:** Business classes (industries) in which the interviewed patients (*n* = 310) were working

Business class	N (%)
Agriculture	30 (9.7)
Industry	76 (24.5)
(Petro)chemicals	19 (6.1)
Food products	17 (5.5)
Metal products	16 (5.2)
Pharmaceuticals	6 (1.9)
Other	8 (5.8)
Building and installation industry^a^	42 (13.5)
Wholesale and retail	30 (9.7)
Cars (incl, repair)	15 (4.8)
Other	15 (4.8)
Transport and storage	23 (7.4)
Accommodation, provision of meal and drinks	27 (8.7)
Health and welfare care	33 (10.6)
Human	27 (8.7)
Veterinary	6 (1.9)
Other	49 (15.8)
Laboratories	18 (5.8)
Waste management	7 (2.3)
Public services (police, etc.)	6 (1.9)
Industrial cleaning	5 (1.6)
Other	13 (4.2)

^a^Involves people working in road construction, house building, painters, plumbers, maintenance technicians, façade and window washers, etc.

Company size varied from 1 to 3000 employees. Most incidents occurred in companies with less than 10 (65 incidents) or 11–50 workers (87 incidents), but there were also 55 incidents in large companies with over 200 employees.

Most victims were employees in a non-management function (60.3%). Supervisors were involved in 11.6% of the incidents. In 9.4% of the incidents the victim was the owner of a one person company and in 1.6% the victim was the owner of a small-sized company (max. 10 employees). Temporary workers, such as side job workers, trainees, holiday or contract workers, were involved in 16.8% of the incidents.

Exposure to hazardous substances can occur at various time points during work: during transport, preparatory activities, actual production or use, repair and maintenance, cleaning, etc. Cleaning activities accounted for more accidents (33.2%) than activities during actual production or use (21.3%). 63.0% of incidents occurring in the business class “Accommodation, provision of meal and drinks” and 37.0% of incidents in the business class “Human health and welfare care”, happened during cleaning activities. Occupational exposures also occurred during preparatory (11.3%) and repair and maintenance activities (14.5%). Only 2.9% of the incidents happened during transport. Thirty-seven victims (11.9%) were not working with the hazardous substance themselves, but were exposed while working in close proximity or just walking by.

### Exposure characteristics

In Table 2 an overview is given of the most commonly involved compounds. Acids and alkalis were most often involved. Most patients were exposed to a liquid (59%), followed by a vapor or aerosol (22.3%), gas (9.4%) or solid or powder (9%). Patients were often exposed via multiple routes, most commonly involving ocular contact (40.0%), followed by inhalation (33.9%), dermal contact (32.6%), and oral exposures (9.4%). Almost 65% of all incidents took place indoors.

**Table 2:** Most commonly involved compounds in occupational exposures reported by the interviewed patients (*n* = 310)

Group	Compound^d^	N
Acids		77
	Nitric acid	13
	Sulfuric acid	8
	Hydrofluoric acid	7
	Hydrochloric acid	7
	Phosphoric acid	6
	(per)acetic acid	6
Alkalis		58
	Sodium hydroxide	33
	Potassium hydroxide	13
Gases		17
	Smoke	3
	Propane	3
	Butane	3
	Carbon monoxide	2
Medicines and vaccines		12
	Vaccine^a^	5
	Pentobarbital/Thiopental^b^	5
Chlorine compounds		25
	Chlorine gas/vapour^c^	16
	Sodium hypochlorite	9
Metals and metal salts		11
	Zinc compounds	3
	Copper compounds	2
Cyclic hydrocarbons		9
	Styrene	3
Alcohols and phenols		26
	Ethanol	13
	Methanol	3
	Isopropyl alcohol	5
Glycols		10
	Ethylene glycol	5
	Propylene glycol	2
Aldehydes and ketones		10
	Formaldehyde	3
	Acetone	4
Fuels		7
	Gasoline/diesel	2
	Natural gas	2
Halogenated hydrocarbons		6
Lubricants		10
	Hydraulic oil	5
Pesticides		12
	Phosphine	3
	Glyphosate	2

^a^3 incidents with COVID-19 vaccine (eye-exposure during vaccine preparation)

^b^5 incidents during euthanizing animals (needle stick injuries)

^c^In 15 incidents chlorine gas was formed during mixture of compounds

^d^Most patients were exposed to a mixture of compounds

### Causes of occupational exposures

Table 3 contains a summary of the root causes involved in occupational exposures. In Supplemental Table 1 more detailed information on the root causes in relation to the specific business classes, is given.

**Table 3:** Most important root causes of occupational exposures reported by the interviewed patients (*n* = 310)

Root causes	N	%
**Technical**		
Damaged packaging	74	23.9%
Defective apparatus	30	9.7%
**Organizational**		
No work instruction	137	44.2%
Poor communication, planning	97	31.3%
**Personal**		
Fatigue, inaccuracy, time pressure, etc.	155	50.0%
Disregarded work instruction	41	13.2%
PPE obligatory, but not used		
Safety glasses^a^	30	9.7%
Protective gloves	6	1.9%

*PPE* Personal Protective Equipment

^a^30 patients did not wear safety glasses, however 3 of them wore a face shield and 5 normal glasses. A face shield was used in all patients in which this was obligatory (*n* = 11)

Technical factors that led to exposure mainly involved damaged packaging and defective apparatus. In 237 cases, the patient used a product that was (initially) packaged. Holes, ruptures or cracks caused 74 incidents. One hundred twenty-two patients used a tool or machine. In 30 incidents a defect occurred, often leakage, clogging and detachment of a hose. When comparing the different business classes, a damaged packaging as cause of the incident was mentioned statistically significant (*p* < 0.05) more often in the business class “Transport and storage” compared to all other business classes (Supplemental Table 1).

The unavailability of a work instruction was often mentioned (44.2%), especially in the business classes “Accommodation, provision of meal and drinks” and “Wholesale and retail.” In addition, 20.3% reported poor communication or poor planning (12.9%). Ninety-six incidents (31.1%) were caused by a mistake made by a colleague instead of the patient him or herself.

With respect to personal factors, 41 workers (13.2%) indicated that they did not follow the instructions. Likewise, obligatory use of PPE was sometimes disregarded. The use of safety glasses was obligatory in 86 patients (27.7%). Nonetheless, 30 of these patients did not wear safety glasses, 22 did not wear any form of face protection, 3 wore a face shield and 5 had their regular glasses on. Less accidents occurred by not wearing protective gloves (*n* = 6). Personal factors such as inaccuracy (*n* = 87, 28.1%), time pressure (*n* = 76, 24.5%) and fatigue (*n* = 27, 8.7%), were often reported. In total 155 patients (50.0%) stated that one or more personal circumstances played an important role in the incident.

### Health effects and treatment

The majority of the patients only reported mild health effects. After oral exposure patients experienced pain in mouth or throat (37.9%) or abdominal pain (20.7%). Ocular contact primarily resulted in pain (59.7%), redness (36.3%) or blurred vision (29.9%). Four patients (3.2%) developed corneal damage. After dermal contact most patients experienced pain (43.6%) and redness (36.6%). More severe effects, such as burns (22.8%) and necrosis (5%), were also reported. Inhalation mainly led to dyspnea (42.9%), coughing (33.3%) and headache (32.4%).

The most commonly used first-aid treatment was rinsing with water after ocular exposure (88.7%), dermal contact (67.3%) or oral contact (72.4%), or leaving the contaminated area (74.3%) in case of inhalation.

The majority of the patients (83.9%) visited a general practitioner. Seventy-five patients (24.2%) were examined or treated in a hospital. Eleven patients (3,5%) were hospitalized for a relatively short period (12–60 h).

Most patients recovered within 1 week (76.5%; 36.9% within 1 day). Sixty-five patients (21%) mentioned absence from work after the incident. Twenty-two patients had an absence period up to 1 day, 39 of 1–7 days and 4 of longer than 1 week.

## Discussion

In the present study, we interviewed 310 patients acutely exposed to dangerous substances at work.

Patients were often exposed via multiple routes (ocular contact 40%, inhalation 34% and dermal contact 33%). A comparable exposure pattern was found in a previous Poison Control Center (PCC) study [7]. Similar to our study, other PCC studies also show that a variety of chemical compounds are involved in occupational incidents, with acids and alkalis ranking high in the number of exposures [7–10].

Most PCC studies describing acute occupational exposures, however, have a retrospective design, focusing on the characterization of the substances involved, specific populations at risk or medical aspects [2, 7–14]. In retrospective studies, information about the circumstances and causes of the exposure is often incomplete or lacking [2, 12]. In our prospective study, by interviewing patients involved in occupational accidents, we show that a Poisons Center is able to collect valuable data on the root causes of these accidents. A better understanding of the technical, organizational and personal factors contributing to accidents, offers opportunities for setting up preventive measures and better worker protection.

Occupational intoxications occur in various sectors of industries. In our study, most incidents occurred in the “Building and installation industry”, “Agriculture”, and “Health and welfare sector.” Incidents happened predominately in small-sized companies. In general, workers in smaller companies have a greater risk for accidents and injuries at the workplace [15, 16]. Temporary employees are generally at higher risk of an accident [15–17]. In this study, temporary workers were involved in 17% of the accidents.

Cleaning is a risky activity, as approximately one third of the accidents occurred during cleaning activities. The Swedish PCC also reported that a substantial part (24%) of occupational incidents involved cleaning agents or disinfectants [18].

When looking at the root causes of occupational intoxications, improper work instruction is an important factor that increases the risk for exposure to hazardous substances. In this study, 44% of the patients reported that there was no work instruction available. This was especially mentioned by patients working in business classes “Accommodation, provision of meal and drinks” and “Wholesale and retail.” Workers that are unaware of the potential hazards of chemicals in their work environment, are more vulnerable to exposure and injury [15, 16]. Therefore, it is important to continuously educate workers on the hazards of the chemicals at work and to provide clear work instructions.

Occupational exposure is not always the fault of the patient him or herself as in 31% of the incidents a patient was exposed to a hazardous substance because a colleague made a mistake. This stresses the importance of vigilance among all employees in a work environment where dangerous substances are used.

Technical factors can cause occupational exposure to dangerous substances. In this study, damaged packaging (especially mentioned by patients working in business class “Transport and storage”) and defective apparatus often caused occupational exposure. These data illustrate that proper maintenance of machinery is important. Instructing employees to careful handle packaging and attention to the design of packages can lead to a further reduction in the number of occupational incidents [19].

Personal factors also play an important role in occupational exposure to hazardous substances. Half of all patients in this study reported that inaccuracy, time pressure and/or fatigue played an important role in the incident. Fatigue increases the risk for injuries at work [16]. Approximately one in three patients did not wear the obligatory safety glasses. In some cases, patients thought a face shield or wearing regular glasses would offer appropriate protection. This shows that merely providing PPE, especially protective glasses, is not enough. In order to decrease the risk of exposure, employers should not only provide but also instruct their employees how and when to use PPE [18, 19]. It should be emphasized that the wearing of PPE is also important during preparatory, maintenance or repair and cleaning activities.

The majority of the patients in our study only reported mild health effects and recovered quickly. This can possibly be explained by the fact that decontamination as a first-aid measure was often carried out promptly after the exposure. Data from other PCC studies also show that the majority of occupational incidents had mild outcomes [7, 12, 13, 20]. One in five patients reported absence from work, mostly during a short period. 80% resumed their normal activities within 5 days. However, it is rather common practice in many companies that mildly affected workers temporarily perform other tasks within the company, until they have fully recovered from their exposure. In general, occupational illness and injury and subsequent absence from work is underreported [15, 16, 21, 22].

In most countries there is a legal obligation to report incidents at work with fatal outcome, hospitalization or permanent injury [15, 16, 23]. This results in robust monitoring of severe, work-related exposures to hazardous substances. Minor injuries requiring first-aid only, are often not reported to governmental authorities. However, small and seemingly insignificant incidents can precede major incidents and in itself offer a chance to learn from these. Because of the easy accessibility the DPIC receives a large number of calls for rather minor health effects from (especially) general practitioners. These incidents are not accounted for in the national labor injury statistics and are therefore considered supplementary to national statistics on work-related accidents. The large number of calls for rather minor health effects from physicians to a Poisons Center reflects the fact that medical professionals, especially general practitioners, have little experience with acute exposure to dangerous substances at work.

PCC data have several limitations that may bias the results. First, data is based on voluntary reports to the DPIC, which may lead to an underestimation of the true incidence of occupational intoxications in the Netherlands. Another potential source of bias is that specific workers might be underrepresented in the study population. For example, workers employed at companies with less progressive occupational protocols may have fear of retaliation or being fired, or temporary workers could be less motivated to participate. A language barrier did not seem to be a major reason for non-participating in our study, as it was only mentioned a few times before the start of the interview. It was remarkable how many patients reported personal factors as an important cause of the incident. Patients may be inclined to falsely attribute the etiology of the accident to certain factors. From our study it remains difficult to judge whether factors such as time pressure or fatigue are solely personal factors or (in part) related to organizational factors. Better defining this would require more in-depth research at a company’s overall organization.

## Conclusions

This study shows that PCC’s can collect valuable data regarding the identification of risk factors in occupational intoxications. These data can be used to improve risk management efforts at the workplace. Combining Poisons Center data with national labor injury statistics, can lead to a more accurate reflection of the true pattern of occupational incidents.

Based on the results of our study, the following recommendations to reduce the risk of acute occupational exposures can be proposed. First, it is important that safety protocols are established for all activities involving hazardous substances. Procedures should be clearly described in work instructions and all personnel, including temporary workers, should be repeatedly educated to fully understand and accurately follow these instructions.

Second, employers should not only provide suitable PPE, but also instruct their employees how and when to use it. PPE should not only be worn during normal working activities, but also during preparation, cleaning, repair and maintenance activities. Third, it is important to acknowledge that personal factors such as fatigue, time pressure and inaccuracy, are major causes of acute occupational intoxications. Reducing the workload of employees working with hazardous substances could diminish this type of incidents.

### Supplementary Information

**Additional file 1.** Questionnaire.

**Additional file 2: Supplemental Table 1.** Most important root causes of occupational exposures related to business class.

## Data Availability

Research data can be shared upon request with the primary author.
