# Correction: Acute occupational exposures reported to the Dutch Poisons Information Center: a prospective study on the root causes of incidents at the workplace

**DOI:** 10.1186/s12995-024-00404-x

**Published:** 2024-02-14

**Authors:** Anja P. G. Wijnands, Irma de Vries, Tim Verbruggen, Maxim P. Carlier, Dylan W. de Lange, Saskia J. Rietjens

**Affiliations:** 1 Dutch Poisons Information Center, University Medical Center Utrecht, Utrecht University, 3508 GA Utrecht, PO Box 85500, the Netherlands; 2 Department of Intensive Care Medicine, University Medical Center Utrecht, Utrecht University, 3508 GA Utrecht, PO Box 85500, the Netherlands

**Correction: J Occup Med Toxicol 17, 19 (2022)**

10.1186/s12995-022-00360-4

In the original version of this article [1], inhalation was mentioned as the most common route of occupational exposure (62%), followed by ocular (40%) and dermal contact (33%). Due to a calculation error, the percentage for inhalation was incorrect. The correct percentage is 34%, i.e. in 34% of patients, occupational exposure occurred via inhalation.

Because of this error, the text of the abstract, results (exposure characteristics) and discussion, should be amended as follows:

**Abstract**: Patients were often exposed via multiple routes (ocular contact 40%, inhalation 34% and dermal contact 33%).

**Results**: Patients were often exposed via multiple routes, most commonly involving ocular contact (40.0%), followed by inhalation (33.9%), dermal contact (32.6%) and oral exposures (9.4%).

**Discussion**: Patients were often exposed via multiple routes (ocular contact 40%, inhalation 34% and dermal contact 33%). A comparable exposure pattern was found in a previous Poison Control Center (PCC) study [7].

The original article has been corrected.
