# Supplementary material for: Acute occupational exposures reported to the Dutch Poisons Information Center: a prospective study on the root causes of incidents at the workplace
Source: J Occup Med Toxicol. 2022 Sep 5;17:19. doi: 10.1186/s12995-022-00360-4 (PMC9441833; doi:10.1186/s12995-022-00360-4)
Supplement: Supplementary file 1 — Additional file 1. Questionnaire. [file 12995_2022_360_MOESM1_ESM.docx]

| **Introduction** | |
| --- | --- |
| Good morning/ afternoon / evening, this is (name interviewer) on behalf of the Dutch Poisons Information Center, University Medical Center Utrecht. Recently, we have been contacted by your physician, to discuss treatment options following an accident at work.  We are currently performing a study to get a better insight into the circumstances of accidents that occur at work. You can help us by answering some questions about how this accident happened. This data is used to prevent these kind of work-related accidents as much as possible in the future.  Your answers will be treated confidentially and the information obtained will be processed anonymously. You can end the interview at any moment or skip specific questions. This is a telephonic interview that will take approximately 15 minutes. The interview will be recorded.  Are you willing to participate in our study?  For patients younger than 16 years old: Do your parents/caregivers agree that you are taking part in this study?  Is this a convenient moment to ask you a couple of questions, or shall I contact you at another moment?  It is important that the questions are answered correctly. Please take your time to think about certain questions and please indicate if a specific question is not clear. | |
|  | |
| **Patient characteristics** | |
| Age | ………………………………………………[years] |
| Gender | - Male - Female |
| What language do you speak? | - Dutch - Fluent Dutch, although I am not native Dutch - A little Dutch, because I am not native Dutch - I do not speak Dutch but I speak English - I do not speak Dutch or English - Another language, namely……………………………………… |
| The interview was not possible due to a language barrier | - Yes - No |
|  | |
| **Description of the incident** | |
| Date of the incident? | ………………………………………………………[day/month/year] |
| Time of the incident? | ………………………………………………………[hour:minutes] |
| Could you please explain (in your own words) what happened. | |
| [free text] | |
| To what substance(s) were you exposed?  Please specify the product(s) and the composition of the product(s). | [free text] |
| Route of exposure?  Multiple answers possible. | - Ingestion - Oral contact (no ingestion) - Ocular contact - Dermal contact, ………[location] …………….[surface area] - Inhalation |
| Type of product (consistency)?  Multiple answers possible. | - Liquid - Nebula/vapor/aerosol - Gas - Solid/powder - Other, namely………………………………………………….. |
| Duration of the exposure? | ……………..……..[duraration in seconds, minutes, hours, days] |
| Did the incident happen inside or outside? Multiple answers possible. | - Inside - Outside |
| In case the incident happened inside, please provide additional information on the space the incident took place. | ………………………………………...[size of the space] ………………………………………...[ventilation yes/no, type] ………………………………………...[other information] |
| Is a concentration in the air determined? | - No - Yes, ………..……….[concentratie] ……………………….[time] ……………………….[detection method] |
| Was there a monitoring system? | - No - Yes, …………………………………..…………....[system type] |
| Did the monitoring system measure an elevated concentration? | - No, because - No monitoring system present - Concentration within normal limits - System defect - Other reason, …………………………………………………... - Yes, ……………………………………………...[concentration] |
| Were there other victims? | - Yes, number of other victims ……………………………[n] - No - Unknown |
|  | |
| **Symptoms** | |
| Did you develop any symptoms? | - Yes [answer the other questions on health effects] - No - Unknown |
| Please explain (in your own words) what symptoms you experienced, when these symptoms developed and how long they lasted. | [free text] |
| Do you think there is a relation between the exposure and the symptoms? | - Yes - No - Unknown |
|  | |
| **Treatments** | |
| Which type of medical professional was consulted?  Multiple answers possible. | - None - General Practitioner - Emergency Department (ED) physician - Hospital physician (other than ED) - Occupational physician - Other, namely…………………………………………………… |
| Was the company in-house emergency service involved? | - Yes, because.….…………………………………………………. - No, because………………………………………………………. |
| Were you treated directly after the incident? | - No - Yes [type of treatment, by whom, time after incident] ……………………………………………………………………… |
| Did you go to an Emergency Department? | - No - Yes [time after incident, duration of stay]   ……………………………………………………………………… |
| Have you been admitted to a hospital? | - No - Yes, length of stay………………………………………… [hours/days] |
| What kind of treatment was performed in the hospital? | - Unknown - No treatment - Yes [kind of treatment, time after incident] ……………………………………………………………………… |
| Did you recover completely? | - Yes - No [remaining complaints] ……………………………………………………………………… |
| How long did it take to recover? | - Recovery within one day - A couple of days, <1 week - 1 week - 1 month - Longer than 1 month |
| Did you have absenteeism? | - Yes,……………………………. [duration of absenteeism] - No |
| Was an occupational physician involved? | - Yes - No |
| Other relevant information on the treatment and the course of the incident? | [free text] |
|  | |
| **Description of the activities at the time of the incident** | |
| What kind of activities were performed when you were exposed? | - Transport - Transhipment - Preparatory work - Normal activities with the product, namely…………………… - Maintenance equipment - Repair equipment - Cleaning - Activities in the vicinity of the incident - Passive present (p.e. walking by) - Other activities, namely………………..……………………… |
| Please describe your activities | [free text] |
| Was there a work instruction available? | - No - Yes [kind of instruction] ………………………..……………… |
| Did you follow the work instruction? | - No, because .....………………………………………………….. - Yes |
|  | |
| **Description of personal protective equipment and protective measures** | |
| Did you wear gloves? | - No - Yes, material…….………………………………………………. - Other, namely……..…………………………………………….. |
| Could the work be done well wearing gloves? | - No, because……………………………………………………… - Yes |
| What did you wear on your head? | - Nothing - Helmet - Cap/Hat - Hoodie - Other, namely..………………………………………………….. |
| Did you use facial protection | - Yes - Safety glasses - Face shield - Other, namely………………………………………………….. |
| Did you use respiratory protection? | - No - Mouth cap - Full face mask - Half face mask - Other, namely..………………………………………………….. |
| Description of the respiratory protection used | [free text] |
| What clothes did you wear: torso and arms? | - Nothing - Overall, material……………………………………………….. - Plastic rain coat - Shirt/blouse covered arms - Shirt/blouse uncovered arms - Sweater/jacket - Winter jacket - Other, namely………………………………………………….. |
| What clothes did you wear: legs? | - Nothing - Short trousers, material…....…………………………………… - Long trousers, material…....…………………………………… - Overall, material……………………………………………….. - Apron - Other, namely………………………………………………….. |
| What did you wear on your feet? | - Nothing - Flip flops/sandals - Rubber boots - High work shoes - Low work shoes - Other, namely………………………………………………….. |
| Was it mandatory to wear protective clothing during the activities you performed? | - No - Yes, ……..………………………………………………..[which] |
| Why did you not wear the protective clothing ? | - Not applicable (protective clothing worn) - Reason..……………………………………………………… |
| Did the protective clothing function adequately? | - Yes - No, reason…….....………………………………………………. |
| Was there some form of ventilation present and did it function adequately? | - No - Yes,……………..…………………………………………[type]   Function adequately:   - Yes - No, reason…………………………………………………... |
| Were there emergency showers available?  If yes, did you use it? | - No - Yes, used - Yes, not used because…….…………………………………… |
| Were there eye showers available?  If yes, did you use it? | - No - Yes, used - Yes, not used because…….…………………………………… |
|  | |
| **Technical factors** | |
| Did you use a machine or special tools? | - No - Yes, answer the following questions |
| Which kind of machine or tools were used? | [free text] |
| What was the age of the machine or tools? | …………………………………………………...[days/weeks/years] |
| Was the machine or tool defective? | - No - Yes, which defect……………………….………………………   How often does the defect occur…….……………………..…  Since when does the defect exist…………………………….. |
| Is the tool or machine regularly checked or maintained? | - No - Yes, frequency…………………………………..………………   When was the last maintenance performed?................……. |
| Has the tool or machine been recently repaired? | - No - Yes, which defect……………………how long ago.………….. |
| Were safety devices on the machine used? | - No - Not applicable (no safety devices present) - Yes, namely.……………………………………..……………… |
| How long have you been working with the tool or machine? | - First time - Longer, ………….…[frequency]…………………..……[since] |
| Was the product you were working with packaged? If so, how? | - No - Yes, ………………………………………...[kind of packaging] |
| Was the packaging damaged? | - No - Yes, ..……………………………[kind and degree of damage] |
|  | |
| **Organizational factors** | |
| What type of company do you work in? | [type of industry, free text] |
| Did the accident take place at the company you work for? | - Yes - No - Other, namely………………..…………………………………. |
| Were you or your company present as a subcontractor on the site where the incident took place? | - Yes - No |
| Are you the owner of the company?  If yes, do you have employees? | - Yes, self-employed with staff ……. [number of employees] - Yes, self-employed without staff - No |
| If you are not the owner, what is your type of employment? | - Employed in a management position (>5 employees) - Employed in a non-management position - Temporary worker - Holiday worker - Contract worker - Trainee, …………………………………….............[education] - Other, namely…………………………….……………………… |
| How many people work in the company? | - Known, namely………………………..[number of employees] - Estimation, <10, 10-20, 20-100, 100-1000 >1000 - Unknown |
| What is your position within the company? | [free text] |
| How long have you been working in this type of industry? | [free text] |
| How long have you been working in this type of function? | [free text] |
| Which education(s) and/or training(s) have you followed? | ………………[kind of education/training] ………………... [when]  ………………[kind of education/training] ………………... [when] |
| Could the incident have been caused by poor communication/appointments/planning?  Multiple answers possible. | - Yes, poor communication - Yes, poor appointments - Yes, poor planning - No |
| If you answered yes to the above question, please explain further. | [free text] |
|  | |
| **Personal factors** | |
| Is it likely that any of the following personal circumstances caused the accident?  If possible, provide additional information  Multiple answers possible. | - Fatigue ………………………………………………………….. - Tension/Stress ..…….………………………………………….. - Rushed work………… …………………………………………. - Inaccuracy ……..…………………………………………….. - Time pressure ………………………………………………….. - Working to long without breaks…………….…………………. - Not enough employees available for the task ………………. - Heavy physical work ………..………………………………….. - Other, namely …………………………………………………… |
| Did you do something wrong yourself? | - Yes, namely……………………………………………………. - No |
| Has a colleague done something wrong? | - Yes, namely……………………………………………………. - No |

| **End of interview** |
| --- |
| This brings us to the end of this interview. I would like to emphasize again that your answers will be treated confidentially.  We appreciate your cooperation and would like to thank you very much for your participation. |
