# Supplementary material for: Acute occupational exposures reported to the Dutch Poisons Information Center: a prospective study on the root causes of incidents at the workplace
Source: J Occup Med Toxicol. 2022 Sep 5;17:19. doi: 10.1186/s12995-022-00360-4 (PMC9441833; doi:10.1186/s12995-022-00360-4)
Supplement: Supplementary file 2 — Additional file 2: Supplemental Table 1. Most important root causes of occupational exposures related to business class. [file 12995_2022_360_MOESM2_ESM.docx]

*Supplementary table 1: Most important root causes of occupational exposures related to business class.*

| **Businessclass*** | **Total group** | **Industry** | **Building and installation industry** | **Health and welfare care** | **Agriculture, forestry and fisheries** | **Wholesale and retail** | **Accommodation, provision of meal and drinks** | **Transport and storage** |
| --- | --- | --- | --- | --- | --- | --- | --- | --- |
|  | N (%) | **N (%)** | **N (%)** | **N (%)** | **N (%)** | **N (%)** | **N (%)** | **N (%)** |
| **Incidents (total)** | 310 | 76 (24.5) | 42 (13.5) | 33 (10.6) | 30 (9.7) | 30 (9.7) | 27 (8.7) | 23 (7.4) |
|  |  |  |  |  |  |  |  |  |
| **Root causes** |  |  |  |  |  |  |  |  |
| ***Technical*** |  |  |  |  |  |  |  |  |
| Damaged packaging | 74 (23.9) | 15 (19.7) | 10 (23.8) | 6 (18.2) | 7 (23.3) | 11 (36.7) | 3 (11.1) | 14 (60.9) |
| Defective apparatus | 30 (9.7) | 7 (9.2) | 4 (9.5) | 2 (6.1) | 6 (20.0) | 5 (16.7) | 1 (3.7) | 1 (4.3) |
|  |  |  |  |  |  |  |  |  |
| ***Organizational*** |  |  |  |  |  |  |  |  |
| No work instruction | 137 (44.2) | 26 (34.2) | 19 (45.2) | 16 (48.5) | 14 (46.7) | 18 (60.0) | 17 (63.0) | 13 (56.5) |
| Poor communication, planning | 97 (31.3) | 26 (34.2) | 13 (31.0) | 11 (33.3) | 9 (30.0) | 7 (23.3) | 9 (33.3) | 9 (39.1) |
|  |  |  |  |  |  |  |  |  |
| ***Personal*** |  |  |  |  |  |  |  |  |
| Fatigue, inaccuracy, time pressure, etc. | 155 (50.0) | 43 (56.6) | 21 (50.0) | 10 (30.3) | 16 (53.3) | 17 (56.7) | 17 (63.0) | 6 (26.1) |
| Disregarded work instruction | 41 (13.2) | 13 (17.1) | 2 (4.8) | 6 (18.2) | 5 (16.7) | 3 (10.0) | 0 | 2 (8.7) |
| PPE obligatory, but not used  Safety glasses | 30 (9.7) | 17 (22.4) | 5 (11.9) | 0 | 1 (3.3) | 1 (3.3) | 0 | 2 (8.7) |
| PPE obligatory, but not used  Protective gloves | 6 (1.9) | 0 | 0 | 0 | 1 (3.3) | 2 (6.7) | 2 (7.4) | 0 |

^a^ The business class “other” (n= 49 incidents) is not mentioned in this table, because this is a very diverse group (see table 1).
